# The OUTREACH study: oncologists of German university hospitals in rotation on a palliative care unit—evaluation of attitude and competence in palliative care and hospice

**DOI:** 10.1007/s00432-022-04131-w

**Published:** 2022-07-13

**Authors:** T. Biersching, A. Schweda, K. Oechsle, F. Nauck, J. Rosenbruch, U. Schuler, J. Hense, M. Neukirchen, M. Weber, C. Junghanss, T. Kramer, C. Ostgathe, P. Thuss-Patience, B. Van Oorschot, M. Teufel, M. Schuler, C. Bausewein, M. Tewes, The OUTREACH Study Group, C. Ostgathe, M. Tewes, S. Gahr, J. Berendt, K. Oechsle, F. Nauck, G. Benze, C. Bausewein, J. Rosenbruch, U. Schuler, J. Hense, M. Neukirchen, J. Schwarz, M. Weber, U. Reinholz, C. Junghanss, T. Kramer, P. Thuss-Patience, B. van Oorschot, C. Roch

**Affiliations:** 1 West German Cancer Centre, Department of Medical Oncology, University Hospital Essen, 45147 Essen, Germany; 2 West German Cancer Centre, Department of Psychosomatic Medicine and Psychotherapy, University of Duisburg-Essen, LVR University Hospital Essen, 45147 Essen, Germany; 3 Palliative Care Unit, Department for Oncology, Haematology and Bone Marrow Transplantation, University Medical Center Hamburg-Eppendorf, Hamburg, Germany; 4 Department of Palliative Medicine, University Medical Centre Göttingen, Göttingen, Germany; 5 Department of Palliative Medicine, LMU Munich Hospital, Ludwig-Maximilians-University, Campus Großhadern, Munich, Germany; 6 University Palliative Care Centre, Carl Gustav Carus University Hospital, Dresden, Germany; 7 Interdisciplinary Centre for Palliative Medicine, University Tumor Centre Düsseldorf - Comprehensive Cancer Centre, University Hospital Düsseldorf, Heinrich Heine University, Düsseldorf, Germany; 8 Department of Anaesthesiology, University Hospital Düsseldorf, Heinrich Heine University, Düsseldorf, Germany; 9 Interdisciplinary Department for Palliative Medicine, University Medicine Mainz, Johannes-Gutenberg-University, Mainz, Germany; 10 Division of Medicine, Dept. of Haematology, Oncology and Palliative Medicine, University Medical Centre, Rostock, Germany; 11 Palliative Medicine at the University Centre for Tumor Diseases (UCT), University Hospital Frankfurt, Frankfurt am Main, Germany; 12 Palliative Medicine Department, Comprehensive Cancer Centre CCC Erlangen-EMN, University Hospital Erlangen, Friedrich-Alexander-University Erlangen-Nürnberg, Erlangen, Germany; 13 University Tumor Centre, Charité University Medicine Berlin, Charité Campus Virchow-Klinikum, Berlin, Germany; 14 Interdisciplinary Centre for Palliative Medicine, University Hospital Würzburg, Julius-Maximilians-University, Würzburg, Germany; 15 German Consortium for Translational Cancer Research (DKTK), Partner Location Essen University Hospital, Essen, Germany

**Keywords:** Palliative rotation, Education, End of life care, Palliative care self-efficacy expectations, Palliative knowledge

## Abstract

**Purpose:**

The effect of the duration of an educational rotation presented at a palliative care unit on the palliative care knowledge gain and the increase of palliative care self-efficacy expectations are unclear.

**Methods:**

This national prospective multicenter pre–post survey conducted at twelve German University Comprehensive Cancer Centers prospectively enrolled physicians who were assigned to training rotations in specialized palliative care units for three, six, or twelve months. Palliative care knowledge [in %] and palliative care self-efficacy expectations [max. 57 points] were evaluated before and after the rotation with a validated questionnaire.

**Results:**

From March 2018 to October 2020, questionnaires of 43 physicians were analyzed. Physicians participated in a 3- (*n* = 3), 6- (*n* = 21), or 12-month (*n* = 19) palliative care rotation after a median of 8 (0–19) professional years. The training background of rotating physicians covered a diverse spectrum of specialties; most frequently represented were medical oncology (*n* = 15), and anesthesiology (*n* = 11). After the rotation, median palliative care knowledge increased from 81.1% to 86.5% (*p* < .001), and median palliative care self-efficacy expectations scores increased from 38 to 50 points (*p* < .001). The effect of the 12-month rotation was not significantly greater than that of the 6-month rotation.

**Conclusion:**

An educational rotation presented in a specialized palliative care unit for at least six months significantly improves palliative care knowledge and palliative care self-efficacy expectations of physicians from various medical backgrounds.

**Supplementary Information:**

The online version contains supplementary material available at 10.1007/s00432-022-04131-w.

## Introduction

Palliative care aims to improve the quality of life of patients with terminal illness and includes the care and involvement of family members, as well as the physical, psychological, social, and spiritual needs of the patient. Regardless of the genesis of the life-limiting illness, palliative care should be available to every patient (World Health Organization (WHO) 2022). To advance the further development of palliative care in Germany, the German Cancer Aid is committed to the expansion of a nationwide care structure and to the further development of Comprehensive Cancer Centers (CCCs) (Deutsche Krebshilfe 2021; Berendt et al. 2016). The national CCC network was organized in 2009 as a network of oncology centers of excellence and currently comprises 14 CCCs, five of which cover several university locations. The CCC network ensures cross-site and cross-sectoral multiprofessional cooperation by all disciplines involved in the treatment of a cancer patient and focuses on translational research and training (Netzwerk Onkologische Spitzenzentren 2016).

The palliative care structure in Germany is very heterogeneous and includes a wide variety of outpatient and inpatient structures (Berendt et al. 2016). Although early integration is recommended in oncology treatment processes, palliative care services are currently still integrated late in the disease trajectory (Alt-Epping 2020; World Health Organization (WHO) 2014;; Haun et al. 2017). The barriers for the palliative care integration could be the insufficient education in palliative care or the lack of transparency of palliative care structures (Alt-Epping 2020). Therefore, it is even more important that resident physicians gain an insight into palliative care and treatment structures early during their board certification training. Previous studies have already demonstrated that palliative care rotations may overcome this barrier, because symptom assessment, medication management, and patient communication about ethically sensitive issues can be improved (Reddy et al. 2019). A first analysis of the German CCC network showed that many CCCs offer rotation programs. A rotation could contribute to earlier integration of palliative care and could create a link between general and specialized palliative care (Berendt et al. 2018). A pilot study at three German CCCs that offered a rotation into specialized palliative care for six or twelve months found that rotating physicians had higher palliative care knowledge, including an overview of palliative care structures, and higher palliative care self-efficacy expectations than did board certified specialists without insight into specialized palliative care (Burmann et al. 2019). The term *palliative care self-efficacy expectations* describes an inner conviction that the caregiver’s individual resources can overcome a critical situation. An adequate reaction to difficult situations requires specific knowledge, and the correct use of this knowledge must be learned. (Pfister et al. 2011).

Thus, the hypothesis arises that a rotation into specialized palliative care improves the palliative care knowledge and the palliative care self-efficacy expectations of oncology residents. Therefore, the aim of the OUTREACH study was to determine the extent to which palliative care knowledge and palliative care self-efficacy expectations change during a rotation on a palliative care unit.

## Methods

### Study design

This multicenter, prospective observational study used a validated questionnaire in a pre–post analysis to determine the effect of a medical rotation presented at twelve palliative care centers of university CCCs. Furthermore, a subgroup analysis compared the effects of six-month and twelve-month rotations and their effects on oncology and non-oncology residents. The physicians answered the same questions at the beginning and the end of the rotation so that the differences in the reported measurements could be attributed to the rotation.

### Instrument – the palliative competence test for physicians

In this study, we used the validated measurement instrument “*Palliative Competence Test for Physicians”*, which measures palliative care knowledge and specific palliative care self-efficacy expectations (Mosich et al. 2017). Our instrument consists of German translations of the questions of the Japanese palliative care knowledge test Palliative care Emphasis program on symptom management and Assessment for Continuous medical Education (PEACE Questionnaire) (Yamamoto et al. 2013) and the questions of the Bonn palliative care knowledge test (Pfister et al. 2011) as specified for the medical profession and supplemented to evaluate the participants’ palliative care self-efficacy expectations. The questionnaire includes eleven items eliciting the characteristics of the rotating physicians and four questions about the structure of the rotation, such as whether pre- and post-rotation conversations took place and whether concrete ideas regarding the rotation were fulfilled.

Knowledge of palliative medicine is elicited by 37 questions about pain therapy, psycho-oncology, palliative-hospice structures, communication, dyspnea treatment, treatment of gastrointestinal symptoms, and ethics. Participants rate their responses on a five-point Likert-type scale (*strongly agree*, *agree*, *disagree*, *strongly disagree,* and *I don't know*). The responses *strongly agree* and *agree,* as well as the responses *disagree* and *strongly disagree,* are summarized in the evaluation. Palliative care self-efficacy expectations are measured by rating responses to 19 questions on a four-point Likert-type scale (*strongly agree*, *agree*, *disagree*, *strongly disagree*). A maximum of 57 points is possible: *strongly agree*, 3 points; *agree*, 2 points; *disagree*, 1 point; *strongly disagree*, 0 points. The self-efficacy expectations section of the questionnaire consists of questions about medical profession, methodological ability, communication competence, and empathy, as well as a question about spirituality.

### Setting and sample

The CCCs provide comprehensive medical care for cancer patients and offer a medical rotation in palliative care units. The inclusion criteria of this study were a license to practice medicine, employment at the CCC, and assignment to a palliative care unit. Physicians with previous experience in palliative care were excluded from participation. Study participants were rotating physicians who were involved in board certification training or who wanted to complete additional certification in palliative medicine.

### Recruitment

Rotating physicians were recruited at twelve German CCCs from March 2018 to October 2020. The recruitment rate was determined by the potential number of rotating physicians at the twelve CCCs and the number of rotating physicians who participated in the study. Potential participants were contacted three times by mail. After the start of the rotation, they had four weeks to confirm their participation in the study. If they did not respond after four weeks, they were counted as non-participants.

### Data collection

Before the start of the rotation, participants received the questionnaire for pre-intervention measurement. The rotating physicians received either personal or email invitations from the head of the palliative care unit or directly from the study coordinator. After providing informed consent, they completed the questionnaire within the first four weeks of the rotation and returned it to the study coordinator. They then received a pseudonymization number. Palliative care rotations lasted for three, six, or twelve months. Four weeks before the end of the rotation, the rotating physicians received the same questionnaire from the study coordinator. To reduce the dropout rate, the rotating physicians were reminded of the post-questionnaire two weeks before and again one week before the end of the rotation. If the post-questionnaire was missing, the rotating physician was counted as a dropout and was removed from the pre–post measurement. Data were collected from March 2018 through September 2021.

### Statistical analyses

Participants were compared for age, sex, religion, years in the profession, specialization, duration of rotation, and board certification (Mann–Whitney U test, Chi^2^ test). For the descriptive analysis, median, standard deviation, range, and percentages were determined with SPSS 26.0. Additional analyses focused on the assessment of change in palliative care knowledge and palliative care self-efficacy expectations, as well as on differential effects of the six- and twelve-month rotations. Furthermore, it was hypothesized that the background of the physician could affect the increase in palliative care knowledge and palliative care self-efficacy expectations, particularly for oncology residents or oncologists as opposed to residents from other medical fields and non-oncologists. Generalized estimated equation models (using R 4.11, R core team, 2021) (R Core Team: R 2018) were constructed with the following variables: rotation duration (six months vs. twelve months), professional background (oncology vs. non-oncology), assessment time (pre-rotation vs. post-rotation), and the interaction between these variables and either palliative care knowledge or palliative care self-efficacy expectations. Generalized estimating equations were used because the dataset lacked normality and homoscedasticity, and because such equations require the fulfillment of fewer assumptions than do mixed linear models (McNeish et al. 2017). Sex and age were also included as covariates. Apart from the specific regression coefficients, Chi^2^ tests were used to detect the global effects of the variable. Statistical significance was set at the level of *p* < 0.05. Additional exploratory analyses (including Fisher’s exact test) were used to describe differential characteristics of physicians with various professional backgrounds who chose to complete a rotation at a palliative inpatient clinic.

Rotating physicians were also given the opportunity to participate in a rotation of only three months’ duration. However, because only three participants chose this short rotation (7% of the data), they were excluded from the main analyses, which compared only physicians who completed a six-month or a twelve-month rotation.

### Ethical approval

The study protocol was approved by the Ethics Committee of the University Hospital Essen (18–8067-BO).

## Results

This multicenter prospective study began in March 2018 and was closed in October 2020, with a total of 72 potential rotating physicians. Of these, 54 decided to participate in the study (recruitment rate, 75%) and completed the rotation, and 43 submitted the post-rotation questionnaire (dropout rate, 20.4%).

The Table 1 provides an overview of the participants’ characteristics. Median age and number of years in the profession were very similar for both the oncology and the non-oncology professional groups. Also similar were the numbers of participants choosing a six-month rotation (21, 49%) or a twelve-month rotation (19, 44%). Nearly half of the participating physicians were board certified (48.8%).

**Table 1:** Characteristics of 43 physicians participating in a palliative care rotation at a German Comprehensive Cancer Center

	Total (*n* = 43)	Oncologists (*n* = 15)	Physicians in other disciplines^c^ (*n* = 28)		*p* value
Age, years					
Median		34	34	34.5	.574^a^
Range		24–44	27–42	24–44	
Sex					.745^b^
Female		26	10	16	
Male		17	5	12	
Professional years					
Median		8	7	8	.609^a^
Range		0–19	2–19	0–19	
Rotation duration					.204^b^
3 months		3	1	2	
6 months		21	10	11	
12 months		19	4	15	
Board certification					.203^b^
Yes		21	5	16	
No		22	10	12	
I aim to work as a palliative care physician					1.00^b^
Yes		30	11	19	
No		10	3	7	

^a^Mann–Whitney *U* test

^b^Chi.^2^ test

^c^Anesthesiology, *n* = 11; psychosomatic medicine, *n* = 2; general medicine, *n* = 4; internal medicine, *n* = 7; other, *n* = 4

### Palliative care knowledge

**Fig. 1:**
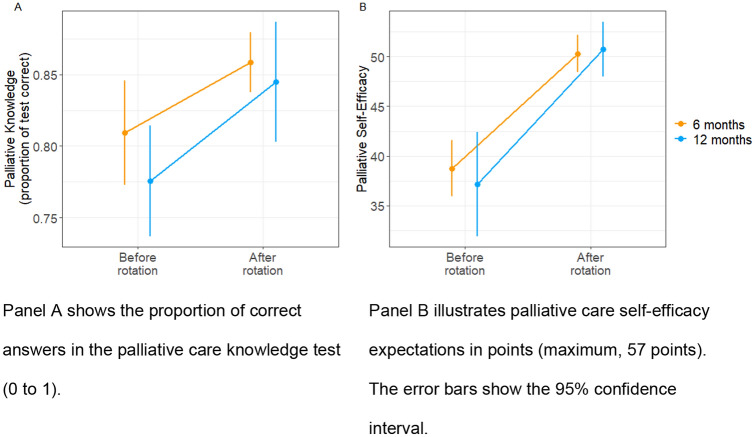
Development of the outcome variables from before the rotation to after the rotation in a palliative care unit (6 months vs. 12 months). Panel **A** shows the proportion of correct answers in the palliative care knowledge test (0 to 1). Panel **B** illustrates palliative care self-efficacy expectations in points (maximum, 57 points). The error bars show the 95% confidence interval

Before the rotation, participants correctly answered a median of 81.1% of the palliative care knowledge questions. After the rotation, the median percentage of questions answered correctly increased to 86.5% (*b* =  − 3.133; *Chi*^2^(1) = 14.31; *p* < 0.001; Fig. 1).

**Fig. 2:**
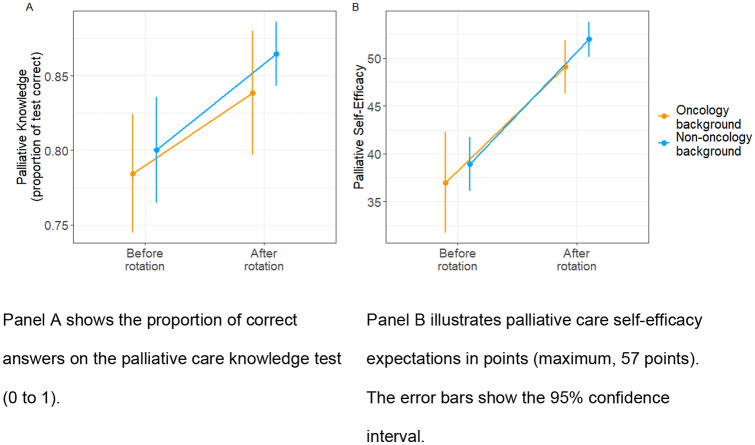
Increase in the outcome variable measurements before and after the rotation according to the professional background of the physicians (oncology vs. non-oncology). Panel **A** shows the proportion of correct answers on the palliative care knowledge test (0 to 1). Panel **B** illustrates palliative care self-efficacy expectations in points (maximum, 57 points). The error bars show the 95% confidence interval

No other significant effect was found. That is, the duration of the rotation did not contribute significantly to the increase in palliative care knowledge (six months: M_pre_ = 0.81, SD = 0.09; M_post_ = 0.86, SD = 0.05; 12 months: M_pre_ = 0.78, SD = 0.09; M_post_ = 0.86, SD = 0.06; interaction rotation duration x time: b = 1.497, Chi^2^(1) = 0.64, *p* = 0.422) (Fig. 1). Also, professional background exerted no group-wise effect on the increase in knowledge (oncologists: Mpre = 0.79, SD = 0.0; Mpost = 0.85, SD = 0.06; non-oncologists: Mpre = 0.8, SD = 0.09; Mpost = 0.87, SD = 0.06; interaction professional background x time = *b* = 1.133, Chi^2^(1) = 0.16, *p* = 0.685) (Fig. 2). Hence, palliative care knowledge increase was equally evident for physicians rotating for six or twelve months and for physicians from different backgrounds. The results were not affected by the inclusion of sex, age, years in profession, and board certification status as covariates (see supplemental material).

### Palliative care self-efficacy expectations

All participants reported significantly higher palliative care self-efficacy expectations after the rotation (M_pre_: 38.0, SD = 7.55; M_post_ = 51.1, SD = 4.76; *b* =  − 15.4, Chi^2^(1) = 82.2; *p* < 0.001). Again, no other regression term exerted a significant effect. The increases were not significantly different for the six-month rotation and the twelve-month rotation (six months: M_pre_ = 38.8, SD = 6.76; M_post_ = 50.3, SD = 4.65; twelve months: M_pre_ = 37.3, SD = 8.42; M_post_ = 51.9, SD = 4.86; interaction duration of rotation x time: *b* = 4.72; Chi^2^(1) = 1.0, *p* = 0.32). Also, the participants’ professional background did not affect the extent of the increase (oncologists: M_pre_ = 38.8, SD = 6.76; M_post _= 49.2, SD = 4.21; non-oncologists: M_pre_ = 38.6, SD = 7.52; M_post_ = 52.1, SD = 4.81; interaction duration of rotation x time: *b* = 3.65; Chi^2^(1) = 0.2, *p* = 0.66). Neither the duration of the rotation nor the participants’ professional background played a role in predicting the increase in self-efficacy expectations during a rotation in a palliative care inpatient clinic. The results were not affected by the inclusion of sex, age, years in the profession, and board certification status as covariates (see supplemental material).

### Differences between medical backgrounds

Of the 43 participants who chose to complete a rotation, most were either oncologists (*n* = 15) or anesthesiologists (*n* = 11). The remaining participants came from general medicine, psychosomatic medicine, internal medicine, and other specialties. Physicians with an oncology background were more likely to opt for a six-month rotation (71.4%) than were physicians with anesthesiology backgrounds (18.2%; *p* = 0.02). Of the participating physicians with an oncology background, only 33.3% were board certified physicians (as compared to residents), whereas participants from anesthesiology departments were mostly board certified (72.7%), but this difference was not statistically significant (*p* = 0.1).

## Discussion

This study found that a significant increase in palliative care knowledge and palliative care self-efficacy expectations can be achieved by a rotation in specialized palliative care. This outcome leads to the optimization of patient care. Neither the participants’ medical background nor the duration of the rotation (six vs twelve months) was of crucial importance. To the best of our knowledge, this is the first national prospective study to analyze the effects of six-month and twelve-month palliative care rotations.

In our study, most rotating physicians had an oncology or anesthesiology background, and previous studies have already shown that these medical specialists play a large role in palliative care (Erlenwein et al. 2014, 2017) According to additional training regulations, basic knowledge of specialist palliative care for patients with systemic diseases and malignant tumors is part of the specific content of the board certification training in internal medicine and in hematology and oncology (Nordrhein 2019). Additional certification for specialist palliative care can be initiated after board certification in any discipline and requires the completion of a 40-h course and an additional 120 h of case seminars, which could be replaced by a six-month internship (Nordrhein 2019). This requirement could provide a hint regarding the lengths of rotations chosen by the various disciplines in our study. Participants in internal medicine or hematology and oncology often choose a six-month rotation as part of their board certification training so that they can gain a deeper insight into palliative care; therefore, these participants are often younger on median than other participants. Anesthesiology physicians rotate after board certification more often than do other physicians as part of an additional qualification as a palliative medicine specialist. As a direct reflection of this fact, compared to anesthesiologists, oncologists at the time of such a rotation have been in the profession for less time and complete board certification training less often.

Our study clearly shows that a palliative care rotation improves palliative care knowledge. La Russa et al. described a deficiency in organized teaching related to palliative care content during medical education. Adequate teaching and supervision regarding the quality of end-of-life care are still lacking for oncology fellows (Russa et al. 2020). Furthermore, studies have shown that no purely didactic teaching method can train physicians comprehensively in palliative care knowledge and that they are not prepared to adequately care for dying patients in an intensive care unit (Kamel et al. 2015). A six- or twelve-month rotation in a palliative care unit could close the described gap in the training of oncology fellows.

The analysis of our multicenter study also detected a significant increase in self-efficacy expectations. Earlier studies using palliative care rotations of various durations have shown that such rotations exert a strong effect on palliative care self-efficacy expectations. A rotation in a palliative care unit should therefore be a mandatory part of medical training (Reddy et al. 2019; Duong and Zulian 2006). Oncologists are often confronted with dying patients and struggle to show adequate distancing and professional behavior in these situations. For this great emotional challenge, a rotation in generalist palliative care is useful in learning important coping strategies (Choo Hwee et al. 2020). Previous research has shown that a one-month rotation improves participants’ ability to deal with pain and symptoms and also strengthens the inner attitude needed for talking with patients about dying and the associated emotions and concerns. (Gunten et al. 1995).

Regarding the duration of the rotation, our results were similar for a six-month and a twelve-month rotation, a finding indicating that even a shorter rotation affects residents’ knowledge and palliative care self-efficacy expectations. Duong et al. previously reported that a six-month rotation in palliative care results in a perceptible improvement in knowledge and in more satisfaction among physicians (Duong and Zulian 2006). Our study also showed that a six-month rotation is not inferior to a twelve-month rotation. Future studies should determine whether shorter rotations equally lead to significant improvement in terms of palliative care knowledge and palliative care self-efficacy expectations.

As part of board certification training, all clinically practicing physicians should gain insight into generalist palliative care of a patient, because almost every medical discipline is confronted with incurable patients and their all-encompassing needs or with patients who are at the crossroads between curative and palliative treatment. The spectrum of diseases has changed dramatically over recent years and is a huge influence on demographic change, so that the treatment of chronic illnesses until the end of life is increasingly becoming the focus (Robert Koch Institut (RKI) 2015). Additional studies and surveys have shown that medical specialists involved in comprehensive oncology care particularly desire an improvement in training regarding end-of-life care and a rotation option in general palliative care. (Mullan et al. 2002; Larrieux et al. 2015; Lester et al. 2011).

## Limitations

One of the limitations of this study is that it was conducted only in Comprehensive Cancer Centers, which are a select group of specialist palliative care institutions. The broad national primary care and rural palliative care structures are not represented here. It must also be noted that the range of all disciplines was limited because of the small sample size. In general, a larger number of rotating physicians is needed for further subgroup analyses. In addition, capturing physicians’ attitudes with a quantitative instrument is challenging; qualitative interviews may provide more insight into the thoughts and experiences of residents. Because our findings clearly show equal improvements irrespective of the duration of the rotation, this short-term effect on residents’ knowledge and palliative care self-efficacy expectation may wane over time; therefore, additional follow-up will be necessary to confirm that six or twelve months of training remain equally effective in the long term.

## Conclusions

Residents rotating to a palliative care unit either as part of their specialist training or for additional development not only indicate its benefit for their medical knowledge but also demonstrate changes in their attitudes irrespective of their medical background. Therefore, we believe that a six-month palliative care rotation should be mandatory in oncology training.

## Supplementary Information

Below is the link to the electronic supplementary material.

Supplementary file1 (DOCX 15 KB)

## Data Availability

The datasets generated during and/or analyzed during the current study are available from the corresponding author on reasonable request.
