# Supplementary material for: The OUTREACH study: oncologists of German university hospitals in rotation on a palliative care unit—evaluation of attitude and competence in palliative care and hospice
Source: J Cancer Res Clin Oncol. 2022 Jul 13;149(7):2929–36. doi: 10.1007/s00432-022-04131-w (PMC10314826; doi:10.1007/s00432-022-04131-w)
Supplement: Supplementary file 1 — Supplementary file1 (DOCX 15 KB) [file 432_2022_4131_MOESM1_ESM.docx]

**Supplemental Material**

**Main analyses controlled for sex and age**

Our study found that residents and board-certified physicians exhibit a statistically significant increase in palliative care knowledge and palliative self-efficacy expectation after their rotation on a palliative care unit. This effect was independent of the duration of the rotation (6 vs. 12 months) and their professional background (oncology vs. other fields). However, such results could be confounded by differential distributions of participants’ characteristics. Therefore, we performed the same analyses but included participants’ age, sex, years of professional experience, and board certification status (yes vs. no) as covariates. Before doing so, we “imputed” two missing data points within the variable age by adding the mean of the age distribution. This was done because of participants’ lack of sociodemographic diversity in terms of income and education. Indeed, the generalized estimated equation models yielded results equivalent to those obtained by the main analyses. The increases in palliative care knowledge and palliative self-efficacy expectation remained statistically significant (both p < .002). However, as was the case with the main analyses, the generalized models found no statistically significant interaction coefficients between the additional variables and the original variables of duration of rotation and professional background. Only sex appeared to modulate palliative self-efficacy: female participants reported higher palliative care self-efficacy expectation overall (b = 3.09; p = .035).
